# Prognostic nomogram for hepatocellular carcinoma with radiofrequency ablation: a retrospective cohort study

**DOI:** 10.1186/s12885-021-08505-0

**Published:** 2021-06-29

**Authors:** Zhenhua Lu, Zhen Sun, Chengyu Liu, Xiaolei Shi, Rui Li, Weiwei Shao, Yangyang Zheng, Yao Li, Jinghai Song

**Affiliations:** 1 Department of General Surgery, Department of Hepato-bilio-pancreatic Surgery, Beijing Hospital, National Center of Gerontology, Institute of Geriatric Medicine, Chinese Academy of Medical Sciences, NO. 1 DaHua Road, Dong Dan, Beijing, 100730 PR China; 2 The Key Laboratory of geriatrics, Beijing Institute of Geriatrics, Beijing Hospital, National Center of Gerontology, National Health Commission; Institute of Geriatric Medicine, Chinese Academy of Medical Sciences, Beijing, PR China; 3 Peking University Fifth School of Clinical Medicine, Beijing, 100730 China

**Keywords:** Hepatocellular carcinoma, Radiofrequency ablation, Nomogram, Cancer-specific survival (CSS), Overall survival (OS)

## Abstract

**Background:**

Radiofrequency ablation (RFA) is an effective treatment option for hepatocellular carcinoma (HCC). This study aimed to analyze the prognostic factors of HCC patients treated with RFA and to develop nomograms for outcome prediction.

**Methods:**

A total of 3142 HCC patients treated with RFA were recruited, and their data were collected from the Surveillance, Epidemiology, and End Results database. Univariate and multifactor Cox analyses were performed to identify independent prognostic factors. These factors were integrated into a nomogram to predict 3- and 5-year cancer-specific survival (CSS) and overall survival (OS). Consistency indices and calibration plots were used to assess the accuracy of the nomograms in both the internal and external cohorts.

**Results:**

The median follow-up periods for HCC patients treated with RFA were 27 and 29 months for OS and CSS, respectively. Marital status, age, race, histological grade of differentiation, tumor size, T stage, and serum alpha-fetoprotein levels at the time of diagnosis were identified as prognostic factors for OS and CSS. Additionally, M stage was identified as risk factors for OS. These risk factors are included in the nomogram. The calibration plots of the OS and CSS nomograms showed excellent consistency between actual survival and nomogram predictions. The bootstrap-corrected concordance indices of the OS and CSS nomograms were 0.637 (95% CI, 0.628–0.646) and 0.670 (95% 0.661–0.679), respectively. Importantly, our nomogram performed better discriminatory compared with 8th edition tumor-node-metastasis (TNM) stage system for predicting OS and CSS.

**Conclusions:**

We identified prognostic factors for HCC patients treated with RFA and provided an accurate and personalized survival prediction scheme.

## Background

In recent years, the incidence of alcoholic and non-alcoholic fatty liver disease has increased due to obesity, alcohol consumption, and environmental factors. Although the incidence of hepatitis B and C is gradually being controlled by vaccines, the incidence of liver cancer has not decreased [1]. Primary liver cancer is currently the seventh most common cancer in the world and the fourth most common cause of cancer-related deaths. The number of cases of liver cancer is expected to continue to rise over the next decade [2]. The histological types of primary liver cancer include intrahepatic cholangiocarcinoma, hepatocellular carcinoma (HCC), and mixed carcinoma. Among them, 75–85% of primary liver cancers are HCCs, which is the main reason for the increase in the number of HCC cases [2]. Minimally-invasive surgery, represented by radiofrequency ablation (RFA), is an important clinical method for the treatment of liver cancer. RFA can generate heat inside tumors to kill cancer cells [3, 4]. Some studies and meta-analyses have shown that for early-stage small HCC, RFA is associated with similar long-term outcomes, shorter hospital stays, and fewer complications as those with liver resection [5]. A study showed an overall median survival of 62 months after RFA for early-to-mid-stage HCC [6]. Studies have also shown that RFA is effective even for tumors measuring 5 cm [7]. For many patients with advanced liver cancer that has metastasized, surgical resection is not recommended because of the massive trauma and poor prognosis. Therefore, RFA is also a better treatment option for advanced liver cancers. However, till date, there have been no clinical studies and statistical tools that evaluated the prognostic factors for patients with different stages of HCC treated with RFA.

The nomogram, a simple and personalized tool based on statistical analysis, calculates the estimated value of each factor to obtain the survival probability of clinical events. It is widely used for disease diagnosis and prognosis [8–10]. In this study, we constructed and validated effective prognostic nomograms to predict cancer-specific survival (CSS) and overall survival (OS) in patients with HCC treated using RFA, to help clinicians provide personalized treatment recommendations.

## Methods

### Data source

The Surveillance, Epidemiology, and End Results (SEER) database is a public database that collects clinical and survival data for 18 population-based cancer registries, covering more than 25% of the U.S. population. This database is updated annually [11]. In this study, the SEER*Stat software (version 8.3.8) was used to obtain patient information from the SEER database.

### Patient choice

Patients were diagnosed with HCC from 2004 to 2015, with the last follow-up in December 2016. The inclusion criteria were as follows: (1) diagnosis of HCC (International Classification of Oncological Diseases: 22.0, histology: 8170–8175), (2) patients who received RFA treatment (SEER code: 16). The exclusion criteria were as follows: (1) unknown cause of death, (2) unknown diagnostic method, (3) aged less than 20 years at diagnosis, (4) unknown TMN stage, (5) unknown tumor size, (6) unknown serum alpha-fetoprotein (AFP) levels, (7) non-primary tumors, (8) survival of patients between 0 and 1 month (death within 1 month is likely to be caused by surgical complications, so it is excluded). Data on clinical characteristics, including marital status, age, race, sex, histological grade of differentiation, diagnostic method, clinical stage, tumor size, metastatic status, 6th edition TNM stage, radiation therapy, chemotherapy, AFP score, fibrosis score, survival time, cause of death, and survival status, were collected from the SEER database. The 6th edition of TNM and clinical staging were converted into 8th edition data through the latest 8th edition of the AJCC staging guidelines [12, 13]. “Vital status codes” and “SEER classification of death from specified causes” were used to set the OS and CSS endpoints.

### Statistical analysis

All patients were randomized in a 7:3 ratio into a training group and a validation group via the R package (‘caret’) [14]. Categorical variables are expressed as frequencies and percentages. The x-tile software was used to define the optional cut-off value of the age and tumor size and further divided into the categorical variables, which was estimated using the Kaplan-Meier method and compared using the log-rank test [15]. Continuous variables of survival time are expressed as medians and interquartile ranges (IQR). In the training and validation groups, categorical variables were compared using chi-square test and continuous variables were compared using Student’s t-test or Mann-Whitney U test (depending on the normality of data distribution and polynomial test correction). Univariate Cox regression analysis was used to evaluate independent survival-related factors in our clinical data. Significant variables (*p* < 0.05) were included in the multiple Cox regression analysis. According to the multivariate Cox regression analysis, variables with *p* < 0.05 were included to construct the 3-year and 5-year OS and CSS prognostic nomograms. Both models were internally validated using bootstrap resampling with 1000 replications in the training cohort and independent external validation in the validation cohort [16]. Harrell’s consistency index (C-index) was used to evaluate the discrimination ability of the prognostic nomograms [17]. Calibration curves were constructed to compare the predicted and observed survival rates. The “RMS” and “survival” packages in the R software (version 4.03) were used for univariate and multivariate Cox analysis and for constructing and validating prognostic nomographs.

## Results

### Patient characteristics

**Fig. 1:**
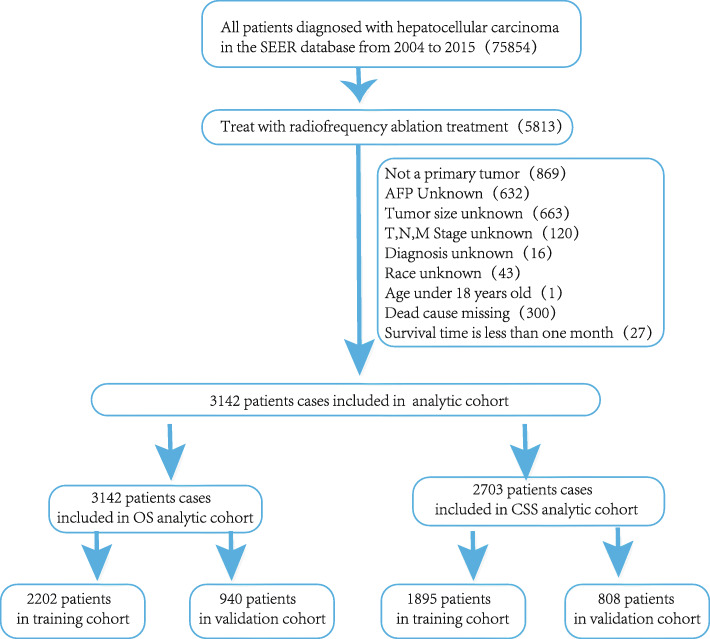
Flow diagram of the hepatocellular carcinoma patients with training and validation cohort

Based on the inclusion and exclusion criteria, this study included 3142 HCC patients treated with RFA in the SEER database from 2004 to 2015. Among them, 439 patients died due to other causes, while the remaining 2703 patients were used for CSS analysis. The data processing flowchart is presented in Fig. 1. In the OS analysis, there were 3142 HCC patients treated with RFA, of whom 2202 were randomly assigned to a training cohort and 940 to a verification cohort. In the CSS analysis, out of the 2703 cases, 1895 were randomly assigned to a training cohort and 808 to a verification cohort. The training cohort was used to construct and internally verify the nomogram, and the verification cohort was used for external verification. Detailed information on the OS analysis of the total, training, and verification cohorts is presented in Table 1, and the detailed information of the CSS cohorts is presented in Table 2. Differences in categorical clinical characteristics between the two groups were determined using chi-square test, with all test *p*-values > 0.05 and no significant differences in demographic or clinical characteristics between the two groups. The median OS and CSS time of HCC patients treated with RFA were 27.00 months (IQR 15.00, 49.00) and 29.00 months [16.00, 50.00], respectively.

**Table 1:** Clinical characteristics of training group and validation group for overall survival analysis

Characteristic	Overall	Training group	Verification group	*P*
	3142	2202	940	
Marital status (%)				
Married	1674 (53.3)	1152 (52.3)	522 (55.5)	0.23
Separated	734 (23.4)	521 (23.7)	213 (22.7)	
Single	734 (23.4)	529 (24.0)	205 (21.8)	
Age (%)				
< 65	2009 (63.9)	1415 (64.3)	594 (63.2)	0.16
65–75	803 (25.6)	545 (24.8)	258 (27.4)	
> 75	330 (10.5)	242 (11.0)	88 (9.4)	
Sex (%)				
Female	762 (24.3)	547 (24.8)	215 (22.9)	0.26
Male	2380 (75.7)	1655 (75.2)	725 (77.1)	
Race (%)				
Other	694 (22.1)	494 (22.4)	200 (21.3)	0.68
White	2070 (65.9)	1440 (65.4)	630 (67.0)	
Black	378 (12.0)	268 (12.2)	110 (11.7)	
Histological grade (%)				
Well differentiated	455 (14.5)	311 (14.1)	144 (15.3)	0.27
Moderately differentiated	541 (17.2)	370 (16.8)	171 (18.2)	
Poorly differentiated	133 (4.2)	103 (4.7)	30 (3.2)	
Undifferentiated	5 (0.2)	3 (0.1)	2 (0.2)	
Unknown	2008 (63.9)	1415 (64.3)	593 (63.1)	
T (%)				
T1a	450 (14.3)	316 (14.4)	134 (14.3)	0.24
T1b	1631 (51.9)	1162 (52.8)	469 (49.9)	
T2	891 (28.4)	601 (27.3)	290 (30.9)	
T3	105 (3.3)	73 (3.3)	32 (3.4)	
T4	65 (2.1)	50 (2.3)	15 (1.6)	
M (%)				
M0	3105 (98.8)	2174 (98.7)	931 (99.0)	0.59
M1	37 (1.2)	28 (1.3)	9 (1.0)	
N (%)				
N0	3092 (98.4)	2173 (98.7)	919 (97.8)	0.08
N1	50 (1.6)	29 (1.3)	21 (2.2)	
Radiation therapy (%)				
Unradiation	3053 (97.2)	2137 (97.0)	916 (97.4)	0.62
Radiation	89 (2.8)	65 (3.0)	24 (2.6)	
Chemotherapy (%)				
Unchemotherapy	2129 (67.8)	1504 (68.3)	625 (66.5)	0.34
Chemotherapy	1013 (32.2)	698 (31.7)	315 (33.5)	
Tumor size (%)				
0-28 mm	1581 (50.3)	1089 (49.5)	492 (52.3)	0.28
21-35 mm	685 (21.8)	483 (21.9)	202 (21.5)	
> 35 mm	876 (27.9)	630 (28.6)	246 (26.2)	
AFP (%)				
Nomal-afp	998 (31.8)	711 (32.3)	287 (30.5)	0.35
Up-afp	2144 (68.2)	1491 (67.7)	653 (69.5)	
Fibersis (%)				
Non-fibersis	171 (5.4)	126 (5.7)	45 (4.8)	0.53
Fibersis	1097 (34.9)	762 (34.6)	335 (35.6)	
Unknown	1874 (59.6)	1314 (59.7)	560 (59.6)	
Survival months (median [IQR])	27.00 [15.00, 49.00]	28.00 [16.00, 50.00]	27.00 [15.00, 48.00]	0.30

**Table 2:** Clinical characteristics of training group and validation group for cancer-specific survival analysis

Characteristic	Overall	Training group	Verification group	*P*
	2703	1895	808	
Marital status (%)				
Married	1438 (53.2)	1020 (53.8)	418 (51.7)	0.423
Separated	623 (23.0)	424 (22.4)	199 (24.6)	
Single	642 (23.8)	451 (23.8)	191 (23.6)	
Age (%)				
< 65	1724 (63.8)	1203 (63.5)	521 (64.5)	0.766
65–75	711 (26.3)	506 (26.7)	205 (25.4)	
> 75	268 (9.9)	186 (9.8)	82 (10.1)	
Sex (%)				
Female	665 (24.6)	465 (24.5)	200 (24.8)	0.945
Male	2038 (75.4)	1430 (75.5)	608 (75.2)	
Race (%)				
Other	617 (22.8)	424 (22.4)	193 (23.9)	0.336
White	1767 (65.4)	1237 (65.3)	530 (65.6)	
Black	319 (11.8)	234 (12.3)	85 (10.5)	
Histological grade (%)				
Well differentiated	389 (14.4)	277 (14.6)	112 (13.9)	0.965
Moderately differentiated	470 (17.4)	324 (17.1)	146 (18.1)	
Poorly differentiated	114 (4.2)	80 (4.2)	34 (4.2)	
Undifferentiated	4 (0.1)	3 (0.2)	1 (0.1)	
Unknown	1726 (63.9)	1211 (63.9)	515 (63.7)	
T (%)				
T1a	388 (14.4)	266 (14.0)	122 (15.1)	0.896
T1b	1390 (51.4)	976 (51.5)	414 (51.2)	
T2	772 (28.6)	543 (28.7)	229 (28.3)	
T3	95 (3.5)	70 (3.7)	25 (3.1)	
T4	58 (2.1)	40 (2.1)	18 (2.2)	
M (%)				
M0	2672 (98.9)	1875 (98.9)	797 (98.6)	0.554
M1	31 (1.1)	20 (1.1)	11 (1.4)	
N (%)				
N0	2658 (98.3)	1865 (98.4)	793 (98.1)	0.731
N1	45 (1.7)	30 (1.6)	15 (1.9)	
Radiation therapy (%)				
Unradiation	2621 (97.0)	1845 (97.4)	776 (96.0)	0.087
Radiation	82 (3.0)	50 (2.6)	32 (4.0)	
Chemotherapy (%)				
Unchemotherapy	1811 (67.0)	1268 (66.9)	543 (67.2)	0.919
Chemotherapy	892 (33.0)	627 (33.1)	265 (32.8)	
Tumor size (%)				
0-28 mm	1377 (50.9)	964 (50.9)	413 (51.1)	0.827
21-35 mm	564 (20.9)	391 (20.6)	173 (21.4)	
> 35 mm	762 (28.2)	540 (28.5)	222 (27.5)	
AFP(%)				
Nomal-AFP	850 (31.4)	603 (31.8)	247 (30.6)	0.551
Up-AFP	1853 (68.6)	1292 (68.2)	561 (69.4)	
Fibersis (%)				
Non-fibersis	151 (5.6)	107 (5.6)	44 (5.4)	0.723
Fibersis	953 (35.3)	659 (34.8)	294 (36.4)	
Unknown	1599 (59.2)	1129 (59.6)	470 (58.2)	
Survival months (median [IQR])	29.00 [16.00, 50.00]	29.00 [16.00, 50.00]	28.00 [15.00, 50.25]	0.55

**Fig. 2:**
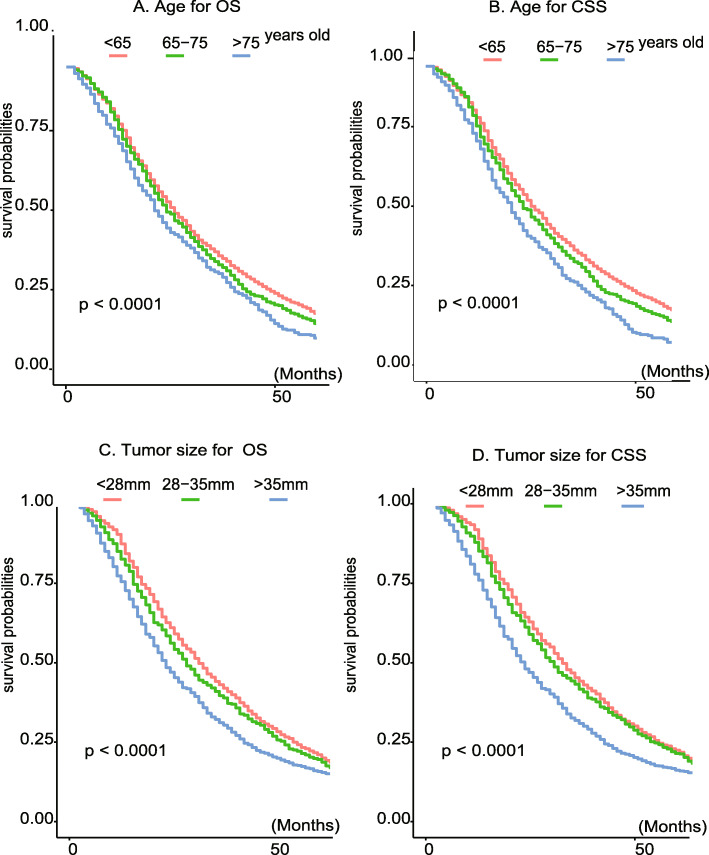
KM survival curve of age group and tumor size in overall survival and cancer-specific survival. **A** KM survival curve of age group in overall survival; **B** KM survival curve of age group in cancer-specific survival; **C** KM survival curve of tumor size group in overall survival; **D** KM survival curve of tumor size group in cancer-specific survival

For tumor size and patient age, we obtained optimal points for continuous variables using the x-tile software. The best cut-off points for age were 65 and 75 years old, and the km survival plots at different ages were separated for OS and CSS, as shown in Fig. 2A and B, the risk increasing with increasing age. The best cut-off points for tumor size were 28 mm and 35 mm, and the survival curves at different tumor stages separated OS and CSS, as shown in Fig. 2C and D, demonstrating that the cut-off points obtained using the x-software were able to distinguish well between high- and low-risk populations. In the entire cohort, 53.3% of the population were married, 63.9% were aged < 65 years, approximately 75.7% were male, and approximately 65.9% were Caucasians. In the 8th version of the AJCC stage group, stage I accounted for 65.1% and stage II accounted for 27.5%. Elevated levels of AFP were noted in 68.2% of all patients. In the combined treatment of the entire study population, more than 32.2% of patients received chemotherapy and only 2.8% of patients received radiotherapy. Lymphatic metastasis occurred in 1.6% of patients, and distant metastasis occurred in only 1.2% of patients.

### Prognostic factors for OS and CSS

A total of 2202 and 1895 HCC patients treated with RFA were included in the OS and CSS analyses, respectively. The results are presented in Tables 3 and 4. In the univariate Cox analyses of OS and CSS, sex, liver fibrosis, chemotherapy, and N stage showed *p* > 0.05, so all other variables were included in the multivariate Cox analysis. Multivariate Cox analysis showed that marital status, age, race, histological grade of differentiation, T stage, M stage, tumor size, and AFP were prognostic factors for OS (*p* < 0.05) and marital status, age, race, histological grade of differentiation, T stage, tumor size, and AFP were prognostic factors for CSS (*p* < 0.05). For T stage, according to the 8th edition AJCC staging standard, we define it as: T1a:The maximum diameter of tumor is ≤2 cm; T1b:Tumor with maximal diameter > 2 cm and no vascular invasion; T2: Tumor with maximum diameter > 2 cm and vascular invasion or multiple tumors (both ≤5 cm in diameter); T3: Multiple tumors, at least one > 5 cm; T4: Tumors, regardless of size, invade the portal vein or major branches of the hepatic vein, or directly invade adjacent organs (except the gallbladder) or penetrate the peritoneum and directly invade other organs.

**Table 3:** Univariate and multivariate cox analysis for overall survival analysis

		Univariate cox			Multivariate cox		
		HR	95%CI	*P*-value	HR	95%CI	*P*-value
Marital status	Married	Reference					
	Separated	1.2	1.05–1.37	0.009	1.19	1.04–1.37	0.011
	Single	1.17	1.02–1.35	0.024	1.24	1.07–1.43	0.003
Age	< 65	Reference					
	65–75	1.16	1.02–1.32	0.028	1.27	1.11–1.46	< 0.001
	> 75	1.71	1.45–2.02	< 0.001	1.87	1.57–2.22	< 0.001
Race	Other	Reference				–	
	White	1.55	1.34–1.79	< 0.001	1.67	1.43–1.94	< 0.001
	Black	1.71	1.41–2.09	< 0.001	1.77	1.45–2.18	< 0.001
Sex	female	Reference					
	male	0.97	0.86–1.11	0.677			
Histological grade	Well differentiated	Reference					
	Moderately differentiated	1.23	1.01–1.5	0.038	1.21	0.99–1.47	0.062
	Poorly differentiated	1.67	1.26–2.2	< 0.001	1.64	1.24–2.17	0.001
	Undifferentiated	24.42	7.76–76.9	< 0.001	29.55	9.22–94.66	< 0.001
	unknown	1.2	1.01–1.41	0.033	1.32	1.12–1.56	0.001
T	T1a	Reference					
	T1b	1.36	1.13–1.63	0.001	1.07	0.88–1.32	0.495
	T2	1.67	1.38–2.03	< 0.001	1.32	1.07–1.63	0.010
	T3	2.86	2.11–3.88	< 0.001	1.53	1.09–2.16	0.014
	T4	4.91	3.47–6.95	< 0.001	2.77	1.90–4.03	< 0.001
N	N0	Reference					
	N1	1.42	0.92–2.18	0.115			
M	M0	Reference					
	M1	2.5	1.67–3.74	< 0.001	1.67	1.09–2.55	0.019
Radiation therapy	Unradiation	Reference					
	Radiation	1.77	1.32–2.39	< 0.001	1.34	0.99–1.83	0.061
Chemotherapy	Unchemotherapy	Reference					
	chemotherapy	1.1	0.98–1.24	0.105			
Tumor size	0-28 mm	Reference					
	28-35 mm	1.34	1.16–1.54	< 0.001	1.31	1.13–1.54	0.001
	> 35 mm	1.85	1.63–2.1	< 0.001	1.64	1.41–1.90	< 0.001
Afp	nomal-afp	Reference					
	up-afp	1.28	1.13–1.45	< 0.001	1.26	1.11–1.43	< 0.001
Fibersis	non-fibersis	Reference					
	fibersis	1.03	0.81–1.3	0.821			
	unknown	1.11	0.89–1.4	0.35

**Table 4:** Univariate and multivariate cox analysis for cancer-specific survival analysis

		Univariate cox			Multivariate cox		
		HR	95%CI	*P*-value	HR	95%CI	*P*-value
Marital status	Married	Reference					
	Separated	1.25	1.07–1.47	0.006	1.25	1.06–1.47	0.009
	Single	1.28	1.09–1.5	0.003	1.35	1.15–1.60	< 0.001
Age	< 65	Reference					
	65–75	1.15	0.99–1.33	0.072	1.26	1.08–1.47	0.004
	> 75	1.79	1.47–2.19	< 0.001	1.98	1.61–2.43	< 0.001
Race	Other	Reference					
	White	1.75	1.47–2.08	< 0.001	1.75	1.46–2.09	< 0.001
	Black	1.5	1.18–1.92	0.001	1.41	1.09–1.81	0.008
Sex	female	Reference					
	male	1.04	0.89–1.21	0.632			
Histological grade	Well differentiated	Reference					
	Moderately differentiated	1.23	0.98–1.54	0.077	1.19	0.95–1.50	0.131
	Poorly differentiated	1.55	1.11–2.18	0.01	1.49	1.06–2.10	0.023
	Undifferentiated	4.19	1.33–13.18	0.014	2.54	0.80–8.10	0.115
	unknown	1.16	0.96–1.41	0.128	1.25	1.03–1.52	0.024
T	T1a	Reference					
	T1b	1.54	1.22–1.94	< 0.001	1.08	0.83–1.39	0.574
	T2	1.94	1.52–2.47	< 0.001	1.39	1.07–1.80	0.013
	T3	3.83	2.73–5.37	< 0.001	1.63	1.11–2.39	0.013
	T4	5.34	3.52–8.11	< 0.001	2.40	1.54–3.73	< 0.001
N	N0	Reference					
	N1	1.49	0.97–2.3	0.071			
M	M0	Reference					
	M1	2.21	1.33–3.69	0.002	1.65	0.97–2.81	0.063
Radiation therapy	Unradiation	Reference					
	Radiation	1.64	1.13–2.38	0.009	1.03	0.70–1.52	0.865
Chemotherapy	Unchemotherapy	Reference					
	chemotherapy	1.1	0.96–1.26	0.168			
Tumor size	0-28 mm	Reference					
	28-35 mm	1.31	1.09–1.56	0.003	1.33	1.10–1.60	0.004
	> 35 mm	2.35	2.03–2.72	< 0.001	2.16	1.83–2.56	< 0.001
Afp	nomal-afp	Reference					
	up-afp	1.42	1.22–1.64	< 0.001	1.44	1.24–1.67	< 0.001
Fibersis	non-fibersis	Reference					
	fibersis	1.05	0.8–1.4	0.709			
	unknown	1.15	0.88–1.51	0.294			

### Construction and verification of the OS and CSS nomograms

**Fig. 3:**
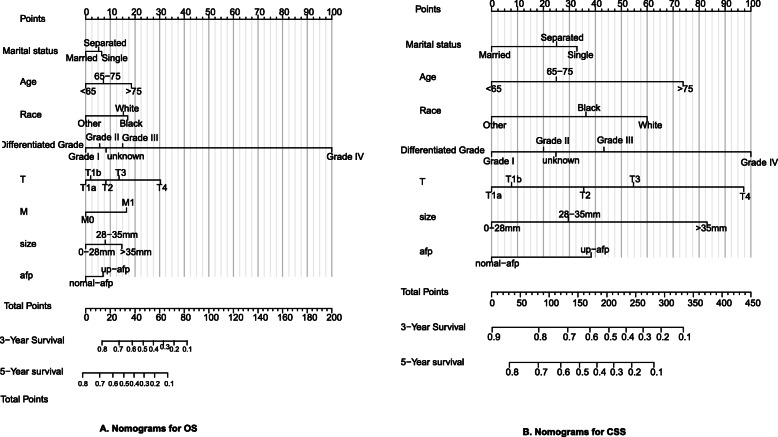
Prognostic nomograms to predict the overall survival (OS) and cancer-specific survival (CSS) of HCC patients with radiofrequency ablation. **A** Prognostic nomograms to predict the overall survival (OS); **B** Prognostic nomograms to predict the cancer-specific survival (CSS)

The above-mentioned prognostic factors of CSS and OS were included to create prognostic nomograms to evaluate the 3-year-and 5-year OS and CSS of HCC patients who underwent RFA (Fig. 3). The nomograms scored each prognostic variable according to the grade and showed that histological grade of differentiation was the main factor contributing to OS prognosis (HR = 29.55 for undifferentiated), followed by age, size, T stage, and M stage. Marital status, race, and AFP score had a moderate impact on OS prognosis. On the other hand, the nomogram showed that differentiation grade, T stage, and tumor size mainly contributed to CSS prognosis, followed by age, and race. Marital status and AFP score had a moderate impact on CSS prognosis. In the nomogram, each factor within these variables was assigned a score on the point scale. By accumulating the total score and locating it on the total point scale, we can easily estimate the probability of 3-year and 5-year survival rates at each score point. For example, a single 30-year-old white female with a 20 mm tumor on her liver with no metastasis detected, no elevated AFP, and a well-differentiated hepatocellular carcinoma on biopsy, treated with radiofrequency ablation. For overall survival, 7 points for her marital status, 0 points for her age, 15 points for her race, 0 points for differentiation, T stage, M stage, size, and AFP. And totaling 22 points. The total points correspond to 3-year survival probability of 73%, a 5-year survival probability of 60%. For cancer-specific survival, 32 points for her marital status, 0 points for her age, 60 points for her race, 0 points for differentiation, T stage, size, and AFP. And totaling 92 points. The total points correspond to 3-year survival probability of 78%, a 5-year survival probability of 68%, which is very convenient for doctors to assess the condition.

### Calibration and validation of the nomogram

**Fig. 4:**
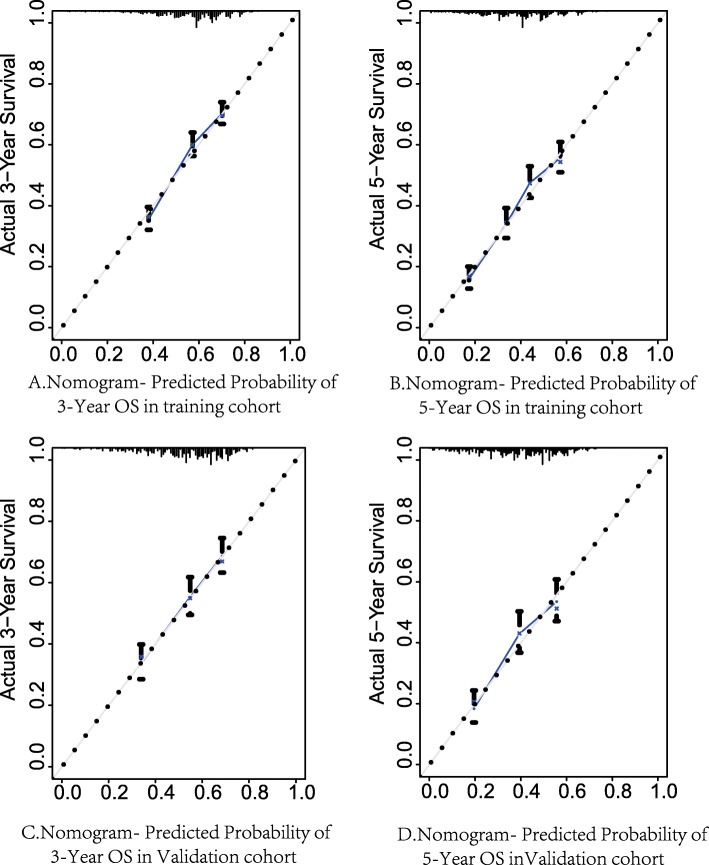
Calibration plots of OS associated nomograms in both training and validation sets. **A** Calibration plots of 3-year OS in training set; **B** Calibration plots of 5-year OS in training set; **C** calibration plots of 3-year OS in validation set. **D** calibration plots of 5-year OS in validation set. OS, overall survival

**Fig. 5:**
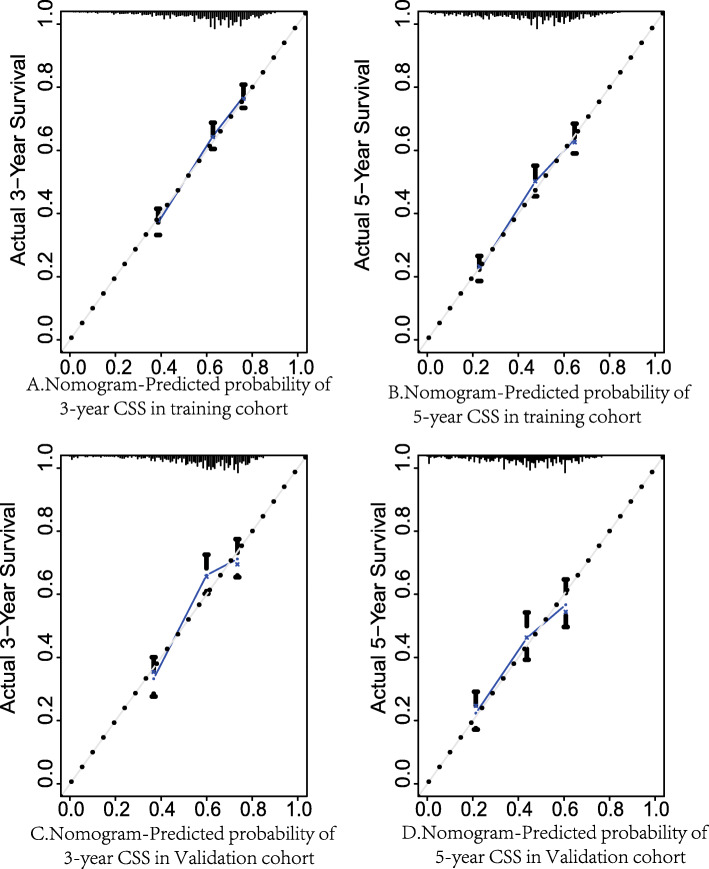
Calibration plots of CSS associated nomograms in both training and validation sets. **A** Calibration plots of 3-year CSS in training set; **B** Calibration plots of 5-year CSS in training set; **C** calibration plots of 3-year CSS in validation set. **D** calibration plots of 5-year CSS in validation set. CSS, cancer-specific survival

Prognostic nomograms were verified both internally and externally (Fig. 4). In the internal verification cohort, the bootstrap-corrected concordance indices of the OS and CSS nomograms were 0.637 (95% CI, 0.628–0.646) and 0.670 (95% 0.661–0.679), respectively. In the external verification cohort, the C-index for OS and CSS was 0.644 (95% CI 0.632–0.656) and 0.666 (95% CI 0.652–0.680), respectively, which were much higher than the C-index for OS [0.576; 95% CI: 0.568–0.584)] and CSS [0.620 (95% CI: 0.606–0.634)] for the 8th edition of the AJCC staging system. On the 3-year-and 5-year calibration plots of OS and CSS (Figs. 4 and 5), the calibration curve did not deviate significantly from the reference line, indicating the accuracy of the prediction model.

## Discussion

RFA is a less invasive and effective treatment method and has important therapeutic significance for treating early HCC and many advanced HCCs. However, there are currently no studies on the prognosis of patients with HCC treated with RFA. Currently, the commonly used clinical evaluation methods are several staging systems, including Barcelona Clinical Liver Cancer (BCLC) and the 8th editions of the American Joint Committee on Cancer (AJCC) staging manuals on tumor, node, and metastasis (TNM) staging systems [18, 19]. However, these staging systems do not integrate overall prognostic factors. The nomogram is a common statistical tool that can predict the survival probability of each patient by including a variety of factors that have an impact on prognosis. However, there is no nomogram for the overall prognosis or CSS analysis of HCC patients treated with RFA.

In this study, we extracted complete information on HCC patients treated with RFA from the SEER database. OS was analyzed in 3142 patients, and CSS was analyzed in 2703 patients. We split the entire cohort into training and validation groups. Differences between the two groups with respect to categorical and continuous variables were tested, and all tests showed *p*-values > 0.05, indicating there was no significant difference between the training and validation groups. To accurately select prognostic factors, we performed univariate and multivariate Cox analyses to identify independent prognostic factors. The multivariate Cox results indicated that marital status, age, race, histological grade of differentiation, T stage, M stage, tumor size, and AFP were prognostic factors for OS, while marital status, age, race, histologic grade of differentiation, T stage, tumor size, and AFP were prognostic factors for CSS.

Marital status is a prognostic factor for OS and CSS in HCC patients treated with RFA. Married patients had longer OS and CSS times than unmarried patients. In the multivariate analysis, even after adjusting for sex, age, race, tumor location, tumor size, pathological grade, and treatment, marital status remained a risk factor for single and separated people. Most single, separated, and divorced cancer patients experience more stress and pain than married patients [20]. In addition, married patients are more likely to comply with treatment, which might lead to better cancer control [21, 22].

Our research also identified age at diagnosis as a risk factor for OS and CSS. Through the x-title software, we suggested 65 and 75 years as the optional cut-off points, which were verified on the KM survival curve. This curve also shows that the prognosis of HCC patients worsens with age. Thus, older HCC patients treated with RFA may have more preoperative comorbidities as the main reason [23]. Tumor size has been most thoroughly studied as a prognostic factor, with many previous studies suggesting 30 mm or 50 mm as the cut-off tumor size [24, 25]. In our study, it was shown that the risk of tumors measuring < 28 mm was not significantly different, but the prognosis of 28–35 mm and > 35 mm was significantly different, and as the size increased, the risk also increased, and the KM curve in the prognosis could be better separated (*p* < 0.05). Therefore, we recommend changing the tumor size’s cut-off to 28 and 35 mm. Besides, AFP levels are elevated in many pregnant women and HCC patients. Currently, it is widely used to diagnose HCC [26, 27]. In this study, we found that AFP was a risk factor for OS and CSS, which is consistent with the results of previous studies [28].

As many HCC patients treated with RFA could not obtain enough tissue for pathological examination, this study did not exclude patients with unknown grades, and the Cox analysis showed that this unknown grade group was ranked between poorly and moderately differentiated tumors. Differentiation had a higher risk ratio for OS and CSS, especially in OS, where the risk ratio was 30 times higher in undifferentiated tumors than in well and poorly differentiated tumors, suggesting that undifferentiated tumors are strongly associated with poor prognosis for OS. This also demonstrates the importance of standardized intraoperative collection of specimens from tumor sites for better understanding of prognosis and better symptomatic treatment [29, 30].

By integrating these prognostic factors, we constructed two nomograms of OS and CSS for HCC patients treated with RFA and verified the nomograms in the training and validation groups; the C-index of the nomograph obtained was significantly higher than that of the 8th editions of the AJCC TNM staging system, which indicates that the nomogram might have better external utility; the calibration curve for the probability of survival showed excellent agreement between prediction by the nomogram and actual observation in the training and validation cohorts. However, this study had some limitations. Although the performance of the nomogram in this study is significant, a multicenter clinical application is needed to evaluate the external utility of the nomogram. Due to the lack of information on liver function and viral infection in the SEER database, more liver function variables should be included in subsequent clinical studies, which may be more accurate in determining the prognosis of HCC patients treated with RFA.

## Conclusions

Our study identified prognostic factors for HCC patients treated with RFA. These prognostic variables were integrated to construct nomograms for determining the prognosis of HCC with RFA. The established nomograms can be used to accurately provide valuable prognostic information, allowing tailed treatments for high-risk patients with HCC with RFA.

## Data Availability

Study data was publicly available in the SEER database (https://seer.cancer.gov/).

## Abbreviations

SEER: Surveillance, Epidemiology and End Results
AJCC: American Joint Committee on Cancer
C-index: Concordance index
CSS: Cancer-specific survival
OS: Overall survival
RFA: Radiofrequency ablation
ICC: Intrahepatic cholangiocarcinoma
HCC: Hepatocellular carcinoma
HR: Hazard ratio
CI: Confidence interval
